# Synergistic China–US Ecological Research is Essential for Global Emerging Infectious Disease Preparedness

**DOI:** 10.1007/s10393-020-01471-2

**Published:** 2020-02-03

**Authors:** Tierra Smiley Evans, Zhengli Shi, Michael Boots, Wenjun Liu, Kevin J. Olival, Xiangming Xiao, Sue Vandewoude, Heidi Brown, Ji-Long Chen, David J. Civitello, Luis Escobar, Yrjo Grohn, Hongying Li, Karen Lips, Qiyoung Liu, Jiahai Lu, Beatriz Martínez-López, Jishu Shi, Xiaolu Shi, Biao Xu, Lihong Yuan, Guoqiang Zhu, Wayne M. Getz

**Affiliations:** 1 One Health Institute, School of Veterinary Medicine, University of California, Davis, CA USA; 2 Wuhan Institute of Virology, Chinese Academy of Sciences, Wuhan, China; 3 Department of Environmental Science, Policy and Management, University of California, Berkeley, Berkeley, CA USA; 4 Key Laboratory of Pathogenic Microbiology and Immunology, Chinese Academy of Sciences, Beijing, China; 5 EcoHealth Alliance, New York, NY USA; 6 Department of Microbiology and Plant Biology, Center for Spatial Analysis, University of Oklahoma, Norman, OK USA; 7 Colorado State University, Fort Collins, CO USA; 8 Mel and Enid Zuckerman College of Public Health, University of Arizona, Tucson, AZ USA; 9 College of Animal Sciences, Fujian Agriculture and Forestry University, Fuzhou, China; 10 Department of Biology, Emory University, Atlanta, GA USA; 11 Department of Fish and Wildlife Conservation, Virginia Tech, Blacksburg, VA USA; 12 Department of Population Medicine and Diagnostic Sciences, College of Veterinary Medicine, Cornell University, Ithaca, NY USA; 13 Department of Biology, University of Maryland, College Park, MD USA; 14 Department of Vector Biology and Control, National Institute for Communicable Diseases Control and Prevention, Chinese Center for Disease Control and Prevention, Beijing, China; 15 One Health Center of Excellence for Research and Training, School of Public Health, Sun Yat-sen University, Guangzhou, China; 16 University of California, Davis, Davis, CA USA; 17 Laboratory of Vaccine Immunology, US-China Center for Animal Health, College of Veterinary Medicine, Kansas State University, Manhattan, KS USA; 18 Department of Microbiology, Shenzhen Center for Disease Control and Prevention, Shenzhen, China; 19 School of Public Health, Fudan University, Shanghai, China; 20 School of Life Sciences and Biopharmaceutics, Guangdong Pharmaceutical University, Guangzhou, China; 21 Jiangsu Co-Innovation Center for Important Animal Infectious Diseases and Zoonoses, Joint International Research Laboratory of Agriculture and Agri-Product Safety of Ministry of Education of China, College of Veterinary Medicine, Yangzhou University, Yangzhou, China; 22 School of Mathematical Sciences, University of KwaZulu-Natal, Durban, South Africa

**Keywords:** China, USA, Emerging infectious diseases, Pandemic, Preparedness, Ecology, Training

## Abstract

The risk of a zoonotic pandemic disease threatens hundreds of millions of people. Emerging infectious diseases also threaten livestock and wildlife populations around the world and can lead to devastating economic damages. China and the USA—due to their unparalleled resources, widespread engagement in activities driving emerging infectious diseases and national as well as geopolitical imperatives to contribute to global health security—play an essential role in our understanding of pandemic threats. Critical to efforts to mitigate risk is building upon existing investments in global capacity to develop training and research focused on the ecological factors driving infectious disease spillover from animals to humans. International cooperation, particularly between China and the USA, is essential to fully engage the resources and scientific strengths necessary to add this ecological emphasis to the pandemic preparedness strategy. Here, we review the world’s current state of emerging infectious disease preparedness, the ecological and evolutionary knowledge needed to anticipate disease emergence, the roles that China and the USA currently play as sources and solutions to mitigating risk, and the next steps needed to better protect the global community from zoonotic disease.

**Electronic supplementary material:**

The online version of this article (10.1007/s10393-020-01471-2) contains supplementary material, which is available to authorized users.

## Introduction

**Figure 1:**
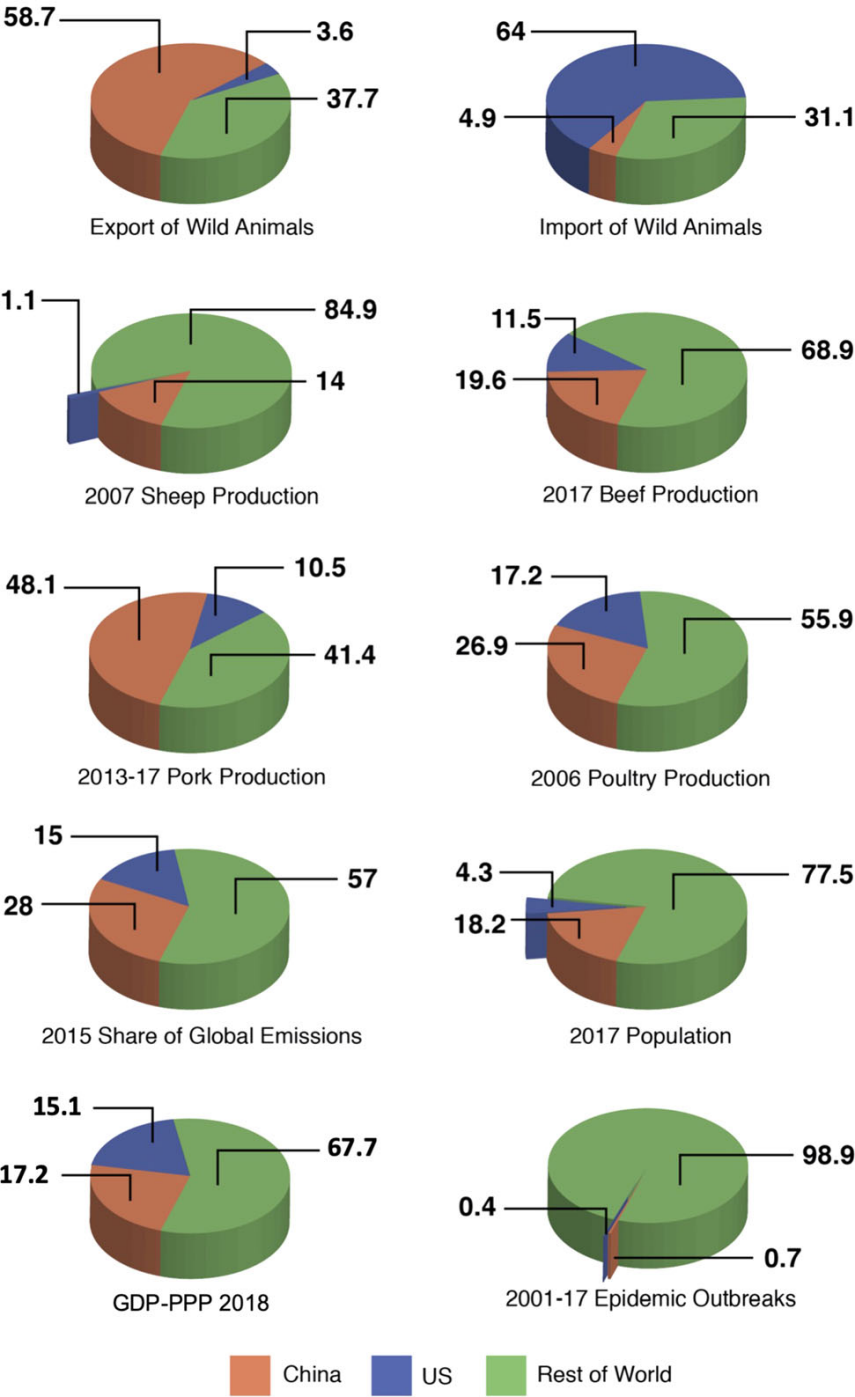
Factors contributing to China, USA and rest of the world’s stake in emerging infectious disease preparedness. (Since values vary with sources and each source updates its estimates from time to time, the actual numbers reported here are not definitive, but should be treated with circumspection.)

The catastrophic health and economic impacts of recent human and agricultural epidemics and the continued risk of novel infectious disease emergence, stemming from the alteration of our planet, are clear (Daszak et al. 2000; Altizer et al. 2011; Allen et al. 2017; Johnson et al. 2015; Carroll et al. 2018). The global nature of emerging and re-emerging infectious disease threats indicates the critical role of international cooperation, particularly spearheaded by China and the USA, in emerging infectious disease (EID) preparedness. China and the USA are well positioned as leaders in the field of EIDs. They are motivated to act out of both national and geopolitical interest and have the resources and instruments to do so: Together they produce over 40% of the world’s livestock (Food and Agriculture Organization of the United Nations 2019; Beef2Live 2019; National Hog Farmer 2019), are the largest legal export/import countries for mammals in the global wildlife trade (Can et al. 2019) and constitute a quarter of the world’s population (Worldometers 2018), and over 30% of the world’s purchasing-power-parity gross domestic product (GDP-PPP) (World Bank 2019) [Fig. 1 (Guha-Sapir 2019; Ritchie and Roser 2019)]. The world would be much better equipped for curbing the next pandemic, if China and the USA provided a united front for research and progress toward EID preparedness. At recent meetings in Shenzhen China and Berkeley California, Chinese and US researchers in the fields of disease ecology, virology, epidemiology, veterinary medicine and public health united to discuss the opportunities for collaboration on infectious disease risk assessment studies that are strongly embedded in ecological and evolutionary principles. What resulted, as discussed herein, is the central role that cooperation between these two highly populous, economic powerhouses is likely to play in understanding the ecological and evolutionary drivers of disease emergence relative to anticipating and managing zoonotic spillovers in China, the USA and worldwide.

## EID Preparedness is Needed

Two hundred years ago, fewer than one billion people inhabited the earth. Today there are over 7.6 billion (Worldometers 2018). This population explosion has necessitated extensive conversion of natural to urban landscapes and agricultural and livestock intensification to support growing human populations. These conversion activities bring wildlife, domestic animals and humans into closer and intensified contact with each other, increasing the likelihood of pathogen evolution, interspecies transmission and spillover of wild and domestic animal pathogens to humans (Daszak et al. 2000; Karesh et al. 2005; Jones et al. 2013). Changes wrought by global warming also play a significant role in pathogen evolution, transmission and spillover, as does expanding socio-economic connectivity—both macro-scale impacts of expanding human populations and resource use. As a result of these global changes, EIDs are on the rise, threatening far-reaching populations (Jones et al. 2008). The economic cost of an EID pandemic today, even in the absence of significant global mortality, is estimated to exceed tens of billions of US dollars (Pike et al. 2014). Understanding the underlying human activities and ecological changes driving EIDs is paramount to preparing for outbreaks and reducing their economic and public health impacts (Barnosky et al. 2012).

The incidence of disease epidemics in wildlife, and pathogens spilling over from wildlife into humans, is increasing in countries where urbanization has recently or is currently occurring (Liu et al. 2018; Hassell et al. 2017). Human urbanization extends city boundaries and alters wildlife host community compositions, leading to biodiversity shifts and loss (Keesing et al. 2010), disturbing the delicate balance between microbes and their natural wild animal reservoir hosts. Human urbanization also brings domesticated animals into closer contact with wild animals, providing opportunities for cross-species pathogen transmission, as evidenced by recent outbreaks of canine distemper virus in giant pandas in China (Feng et al. 2015; Jin et al. 2017) and the introduction of feline leukemia virus in the Florida puma in the USA (Cunningham et al. 2008). Changes in wildlife populations due to infectious disease outbreaks can have long-term serious consequences for ecosystem resilience. Disease outbreaks in wildlife may also result in direct infection of nearby human populations or changes in wildlife host population dynamics, leading to spillover of other pathogens to humans or domestic livestock (Daszak et al. 2000). A prime example is the recent emergence of a novel coronavirus, SADS-CoV, from bats to pigs, resulting from pig farms encroaching on bat habitat, devastating several pig farms in Southern China and posing a major biosecurity risk to the USA (Luo et al. 2018).

The rise in intensive farming, particularly in China and the USA, also potentially creates conditions for selection of more virulent pathogens and greater opportunities for cross-species transmission (Mennerat et al. 2010). China has recently experienced a rapid transformation from small-scale farms to large-scale livestock production enterprises. During 2018, pork production throughout China was estimated at 5404 tons, with the number of individual slaughtered hogs estimated at 693,820,000 (The National Bureau of Statistics 2018a). The number of slaughtered poultry in 2016 was estimated to be greater than 12.3 trillion (The National Bureau of Statistics 2018b). Such intensive production practices in China have contributed to outbreaks of influenza H5N1, H1N1, H7N9, and African Swine Fever viruses (Wei et al. 2016; Wang et al. 2018). In the USA, due to high-density livestock production methods, there has been identification of methicillin-resistant *Staphylococcus aureus* (MRSA) strains in people living in proximity to these farms (Casey et al. 2014).

China is the largest producer and user of antibiotics in the world (Qiao et al. 2018), increasing the likelihood of AMR pathogen development in food-borne illness. China was estimated to use 162,000 tons of antibiotics in 2013 (48% by humans and 52% by animals, respectively) which was 9 times that used in the USA in 2011–2012 (Zhang et al. 2015). Approximately 46% of the antibiotics used in China were ultimately released into rivers through sewage effluent with the remainder distributed on land through manure and sludge spreading (Zhang et al. 2015). AMR is particularly acute in China because of its over-prescription and self-administration practices, as well as its widespread misuse of sub-therapeutic doses of antibiotics in the livestock industry (Yezli and Li 2012; Yu et al. 2014). Antimicrobial-resistant *Escherichia coli* (plasmid-mediated colistin resistance mechanism, MCR-1) originating from overcrowding and high-intensity farming of pigs in China (Liu et al. 2016) can at present be found in countries far from China, including the USA (Hu et al. 2016; Skov and Monnet 2016; Sun et al. 2018). China and US societies are now integrally connected, with intensive regional and international human movement (Tatem et al. 2006), trade of domestic and wild animals (Marano et al. 2007), and other economic activities facilitating the spread of infectious pathogens from high-density farming operations to other geographic locations.

Increasingly complex and robust global trade systems are also fueling the legal and illegal wildlife trade markets. The USA is the largest importer, and China is the largest exporter of legally traded wild mammals (Can et al. 2019) and while challenging to quantify, China is considered the leading country in the consumption and illegal trade of wildlife (Karesh et al. 2005; Patel et al. 2015). Southern China is a hub for domestically and internationally imported and exported wildlife given its strategic geographic location near major ports of trade, dense human population and increasing human mobility. Local tradition also fuels consumption of wild animals in this region. In Guangdong province alone, there are more than 1300 enterprises engaged in wildlife breeding, of which approximately 600 breed wild animals regulated by CITES Appendix II. During 2001–2004, a total of 21 bird species (around 56 thousand individuals), 21 mammal species (~5400 individuals), 41 amphibious and reptile species (up to 346 tons) were raised and traded in Guangzhou, the most populous city in Guangdong Province. Communities engaged in this concentrated wildlife production and trade enterprise represent a valuable resource at the front lines of pathogen spillover and can be leveraged to understand and control the spread of zoonotic diseases.

At a macro-scale, anthropogenic activities have influenced microbial transmission dynamics, particularly for vector-borne pathogens (Jones et al. 2008; Goklany 2009; Woodward et al. 2014; Murray and Daszak 2013; Morse 1995). For example, climate variation has been documented to drive transmission of dengue virus dynamics in Guangdong, China (Liu et al. 2018; Xu et al. 2017; Sang et al. 2014, 2015; Xiang et al. 2017). This region experienced no outbreaks of dengue-like illness from the period of 1950–1977, followed by a relatively low level of incidence until a large-scale outbreak occurred in 2014, infecting over 45,000 people (Liu et al. 2018). Similarly, climate change is projected to impact the distribution of vector-borne disease in the USA, with the environment being more suitable for the introduction of Zika virus in certain regions of the southeastern USA (Carlson et al. 2018). The combined US–China resource capacity is critical to better understand and mitigate anthropogenic EID drivers, such as urbanization, biodiversity loss, landscape conversion, intensive farming and climate change that are contributing to the spread of disease across the wildlife–livestock–human health continuum.

## Required Ecological and Evolutionary Perspective

A holistic ecological and evolutionary process perspective is required to understand the risk of spillover and spread of pathogens in humans and animals (Alexander et al. 2018). Pathogens are not fixed entities, and some pathogens carry a greater innate ability to evolve and spillover into new hosts than others (Johnson et al. 2015; Olival et al. 2017). Evaluation of pathogen evolution from initial spillover to establishment in the human population (e.g., simian immunodeficiency virus chimpanzee to human immunodeficiency virus; HIV-1) (Gao et al. 1999) is critical to understanding why certain pathogens can establish and others cannot. Viral surveillance in “real-time” is required to examine and track pathogen evolution. For example, the Global Influenza Surveillance and Response System, designed to monitor the quickly evolving and recombining influenza virus in a timely manner, has served for over half a century as a global alert mechanism for the emergence of influenza viruses with pandemic potential (World Health Organization 2019a). Knowledge of ecological scenarios surrounding the accelerated viral evolution of highly pathogenic avian influenza (HPAI) is vital to preparing for future outbreaks of the disease in humans and worthy of significant investment in China and elsewhere (Gao 2018). Monitoring of viral evolution in similarly relevant time scales for other quickly evolving pathogens, such as coronaviruses, lags further behind and is an area of research with much needed additional attention. For example, recent evolutionary analyses of Middle East Respiratory Syndrome (MERS) coronavirus have helped to elucidate viral transmission dynamics between camels and people (Dudas et al. 2018). We also now know that viruses sharing diverse vertebrate hosts are “worth watching” for potential emergence in humans, because host breadth (i.e., infecting a taxonomically diverse range of hosts) is a key factor associated with a virus’ likelihood of spillover and secondary human-to-human transmission and geographic spread (Johnson et al. 2015; Olival et al. 2017). Thus, expanding “real-time” surveillance of such pathogens would be a worthwhile investment for public health.

Analyzing epidemiological data using theory and methods from macroecology allows us to forecast impacts of ecological and environmental drivers on infectious disease incidence. Examples include how land cover and climate changes influence epidemics in wildlife (e.g., chytrid fungus, white-nose syndrome), livestock (e.g., foot-mouth-disease, African swine fever, bluetongue) and human (e.g., schistosomiasis, malaria) populations, as well as impact complete wildlife–livestock–human systems (e.g., avian influenza, tuberculosis) (Estrada-Pena et al. 2014; Peterson 2014; Purse et al. 2005). Large-scale, high-resolution data on environmental and climatic factors and populations of humans and wildlife facilitate the creation of dynamic distribution models for infectious agents and can help prioritize research and control efforts (Cohen et al. 2016; Carlson et al. 2017). Collaborative research between China and the USA could greatly advance spatiotemporal disease prediction schemes because both countries encompass large climatic gradients, have high capacity for ecological and climatic data collection, and share some overlapping vectors, and zoonotic pathogens (Liu et al. 2018; Estrada-Pena et al. 2012; Springer et al. 2015; Wu et al. 2013; Centers for Disease Control 2018).

A complete understanding of host–pathogen interactions and how and where to intervene requires an eco-system or “One Health” viewpoint that accounts for processes occurring at both macro- and micro-scales, including at the pathogen, host and environmental levels, as well as an integration of the effects of processes across these scales (Alexander et al. 2018; Forst 2010; Blackburn et al. 2019). Such multiscale “One Health” research requires the incorporation of many disciplines including, but not limited to, human medicine, veterinary medicine, public health, environmental science, ecology, conservation biology, nursing, social sciences, the humanities, engineering, economics, education and public policy (Lu et al. 2016; Carlson et al. 2018). China and the USA have led response activities for several epidemic and pandemic outbreaks impacting humans and animals which have required a One Health perspective. Three relevant case studies which are expanded upon below include: the West Africa Ebola outbreak, the SARS pandemic, and the emergence of amphibian chytridiomycosis.

**Figure 2:**
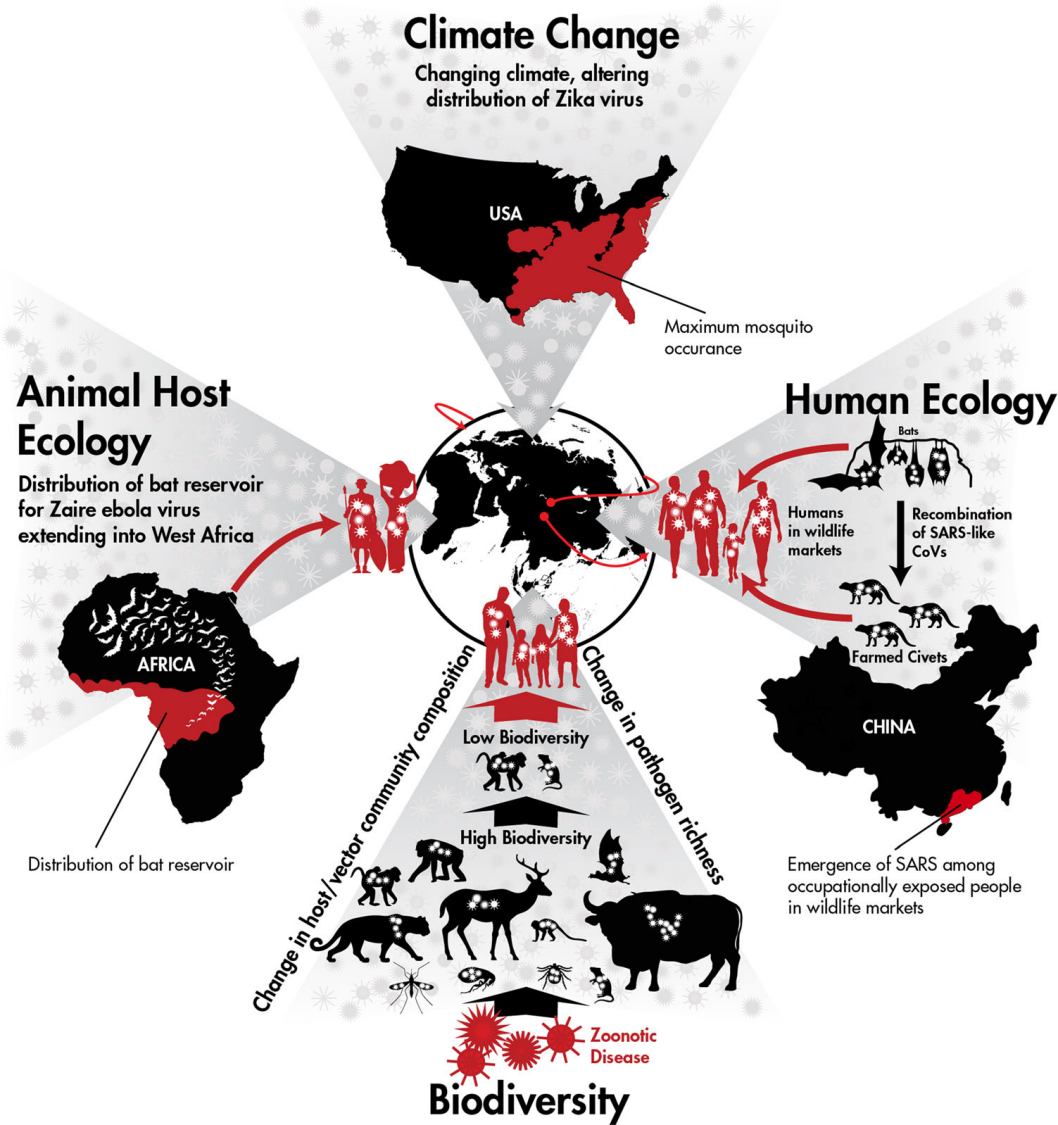
One Health concepts impacting emerging infectious diseases. Climate Change: With the introduction of Zika virus into the Americas, changes in maximum occurrence of mosquito vectors in the USA, due to a changing climate, impact risk of Zika virus distribution. Human Ecology: High-risk human behaviors involving contact with farmed wild animals contributed to the emergence of SARS. Biodiversity: Alteration of wild animal reservoir host populations impacts spillover risk for zoonotic infectious diseases. Animal Host Ecology: Distribution of the bat reservoir hosts for Ebola virus (species *Zaire ebolavirus*) likely caused the first human outbreak in West Africa. Together with the impact of global trade and travel, these case examples of the interconnectedness of humans, animals and the environment demonstrate how human and animal ecology influence the global spread of disease.

The first outbreak of Ebola virus disease (EVD) outside of Central Africa demonstrated the importance of focusing on wildlife host and human ecological risk factors in advance of major disease outbreaks and the need for international collaboration in outbreak response (Fig. 2). Despite nearly 40 years of research since the first outbreak in 1976, including public investment of US$ 1.035 billion between 1997 and 2015 (Fitchett et al. 2016), national and international public health agencies were caught off guard by the 2014 outbreak in West Africa (Kamradt-Scott 2016) that resulted in 28,600 cases with more than 11,300 deaths (WHO Ebola Response Team et al. 2016). Evidence from humans and wildlife indicating the distribution of Ebola virus (species *Zaire ebolavirus*) in West Africa existed prior to this health crisis. Distribution and migration patterns of the hammer-headed fruit bat (*Hypsignathus monstrosus*), little collared fruit bat (*Myoncycteris torquata*), straw-colored fruit bat (*Eidolon helvum*) (Leroy et al. 2005), Franquet’s epauletted fruit bat (*Epomops franqueti*) (Olival and Hayman 2014) and the greater long-fingered bat (*Miniopterus inflatus*) (Kupferschmidt 2018), species implicated as reservoir hosts for Ebola virus, were known to extend into West Africa with opportunities for spread of the virus. Human serological exposure also indicated a wider geographical range for ebolaviruses including Guinea (Boiro et al. 1987), Liberia (Van der Waals et al. 1986) and Sierra Leone (Schoepp et al. 2014) well in advance of 2014. During this outbreak, China and the USA collaborated together for the first time in an international health emergency outside of their borders, which constituted China’s largest ever humanitarian mission in addressing a public health emergency of international concern (Huang 2017). Twenty-four of the Chinese public health experts who were deployed to Africa were graduates of, or residents in, the Chinese Field Epidemiology Training Program (CFETP) established by the US Centers for Disease Control and Prevention (CDC) (Centers for Disease Control 2016). Also, the Chinese government sent 115 military medical professionals to Sierra Leone to work along with US medical personnel to assist with infection prevention and control, clinical care and health promotion and training (Lu et al. 2016). Further investment from China and the USA in working together on response efforts will undoubtedly be mutually beneficial for pathogens of importation concern. Efforts toward unraveling the disease ecology of ebolaviruses—including a better understanding of the ecology of reservoir host(s), the role of secondary spillover hosts, as well as human behaviors surrounding exposure—is also needed and would benefit exponentially from a China–US collaborative effort.

The Severe Acute Respiratory Syndrome (SARS) pandemic caused by a novel zoonotic coronavirus (SARS-CoV) was the first pandemic of the twenty-first century and spread to more than 30 countries (Fig. 2). Initial isolation of SARS-related coronavirus (SARSr-CoV) from masked palm civets and the detection of SARS-CoV infection, in humans working at wet markets selling these animals in Guangdong Province, suggested that masked palm civets could serve as a source of human infection (Guan et al. 2003). Subsequently, SARSr-CoVs were detected in Chinese horseshoe bats (*Rhinolophus sinicus*) and provided strong evidence that bats are the natural reservoir of SARS-CoV (Ge et al. 2013; Li et al. 2005; Yang et al. 2015). Through long-term human and wildlife surveillance, investigators from China and the USA subsequently found that bats carry a diverse range of SARSr-CoVs (Ge et al. 2013; Li et al. 2005; Yang et al. 2015; Lau et al. 2005; Drexler et al. 2010; Yuan et al. 2010; He et al. 2014; Wu et al. 2016; Hu et al. 2017), extending as far as Yunnan Province, and some of them can directly infect humans without intermediate hosts (Wang et al. 2018). While it is unknown whether the SARS outbreak could have been preempted with this knowledge, the joint efforts of China and the USA to rapidly determine where, how and when the virus was spilling over and what human behaviors and populations were at greatest risk for infection may have reduced the severity of the outbreak and will help in mitigating future spillover events. The SARS outbreak was a prime example of the importance of contextualizing epidemiologically notable human behaviors in social, economic and cultural systems in order to decipher causality of an EID.

Pandemic diseases in wild animals (epizootics) can also result in devastating impacts to a country’s biodiversity and natural resources. For example, amphibian chytridiomycosis, caused by the novel pathogen *Batrachochytrium dendrobatidis*, is responsible for massive losses of biodiversity to an entire Class of organisms (Amphibians). The global pandemic lineage of the pathogen originated in Eastern Asia (Ostfeld and Keesing 2012) and disseminated through human trade and transportation (O’Hanlon et al. 2018) into the biodiverse areas of Australia and the Neotropics. The lack of demographic studies, combined with limited population estimates in the IUCN Red List prior to this pandemic, made it difficult to understand the scope of the disease. A better understanding of wildlife population dynamics before massive declines occur is essential to better understanding biodiversity’s impact on EIDs and what elements of biodiversity disease theory (Fig. 2) apply (Jones et al. 2008; Keesing et al. 2010; Murray and Daszak 2013; Morse 1995; Ostfeld and Keesing 2012; Civitello et al. 2015). Interestingly, another fungal pathogen, white-nose syndrome (WNS), which has decimated bat populations in the USA, may also have an Asian or Eurasian origin because it has been found on bats in Northern China (Hoyt et al. 2016). Recent studies suggest that the systemic effects of WNS may down-regulate anti-viral responses in bats persistently infected with coronaviruses and increase the potential of virus shedding (Davy et al. 2018). Thus, a pathogen predominantly infecting wildlife may additionally have cascading effects on spillover of other pathogens of significance for human health.

## Current State of Readiness

Global-scale, government-sponsored EID preparedness efforts, initiated or supported to date by joint China–US partnerships, have focused on improving early-warning capabilities for known and novel human pathogens in both humans and animal reservoirs. These initiatives have included the US CDC Global Disease Detection program (CDC-GDDP), which has collaborated with China CDC and 10 other nations to develop international centers that help countries prevent, detect and respond to public health threats (Centers for Disease Control 2018); the US Agency for International Development’s (USAID) Emerging Pandemic Threats (EPT) Program, which has collaborated with China CDC and Wuhan Institute of Virology and 29 other nations to strengthen EID preparedness through pathogen surveillance in wildlife, domestic animals and humans, risk characterization for pathogen spillover and One Health training and outreach; the WHO, OIE and FAO’s collaborative global early-warning system for animal diseases transmissible to humans (GLEWS) (World Health Organization 2018); the China National Global Virome Initiative (CNGVI; part of the Global Virome Project (GVP Carroll et al. 2018), a pathogen discovery project proposed to identify a large portion of the remaining undiscovered viruses (Carroll et al. 2018; Mora et al. 2011; Geoghegan et al. 2016); and joint research supported by the US National Institutes of Health (NIH) to define the origin of SARS- and MERS-like coronaviruses (Luo et al. 2018; Hu et al. 2017) and identify other SARS coronavirus mammalian infections in China (Zhou et al. 2018).

Over the past ten years, the USA has made a significant investment in spear-heading an international network of government collaborative laboratories and surveillance mechanisms for EID preparedness through the USAID EPT program, CDC-GDDP and the Department of Defense’s Overseas Research Laboratories. For example, the PREDICT project, a part of the EPT program, was operational in 30 countries, increasing capacity in over 60 laboratories located in EID hot spots and training over 6200 health professionals in laboratory diagnostics, field epidemiology, surveillance and biosafety (PREDICT Consortium 2019). Such a large-scale investment by the USA in global disease surveillance, targeting both humans and animals, has laid the foundation and built the networks and infrastructure necessary for implementing future training and research in the underlying disease ecology of EIDs.

China, with its much larger population, has historically taken a more nationalist perspective toward EID research and preparedness. Following the outbreak of SARS in China, the Government enhanced infectious disease surveillance, building the web-based Nationwide Notifiable Infectious Disease Reporting Information System (NIDRIS) (Yang et al. 2017). Based on experience detecting and responding to national epidemics such as SARS, and influenza H5N1, H1N9 and H7N9, China has expanded their efforts to assist with diagnostics and surveillance in the region; they are a participant in the Mekong Basin Disease Surveillance Network (Phommasack et al. 2013) and committed to promoting the prevention and control of communicable diseases and public health emergency response through ASEAN (Association of Southeast Asian Nations)–China health cooperation (Association of Southeast Asian Nations 2018). The China International Development Cooperation Agency (CIDCA) (Chinese International Development Cooperative Agency 2019) has historically invested in infrastructure projects but is now increasingly supporting global health initiatives.

## China–US Leadership

China and the USA are well placed to lead efforts in EID preparedness both from a national interest standpoint, resource availability and a global health interconnectedness perspective. China and the USA have a long history of collaboration, have the two largest economies in the world with significant resources for investment in global health, the largest current combined investment in infrastructure for infectious disease research, and have the skill sets necessary for advancing disease prevention and response. China and the USA first signed a Protocol for Cooperation in the Science and Technology of Medicine and Public Health in the 1970s (Obamawhitehouse.gov. 2018). Today, more than 40% of publications from Chinese scientists are co-authored with scientists from the USA (Wang et al. 2013). With successful poverty reduction in China and transformation from a recipient to a provider of aid, China has taken a more active role in global health initiatives, signing an MOU with the USA in 2017 to designate funding toward cooperation on international development, focusing on food security, public health, humanitarian assistance and disaster response (Carnegie-Tsinghua Center for Global Policy 2018). China has also made efforts to streamline international scientific collaborations, issuing from the General Office of the State Council in March 2018, the regulatory document entitled, “*Measures for the Management of Scientific Data*” (People’s Republic of China 2018; Uga et al. 2001), expected to standardize the data sharing process with the goal of encouraging collaboration.

Since the end of the nineteenth century, the USA has maintained the largest economy in the world and has been the preeminent international influence on global trade and foreign direct investment (International Monetary Fund 2018). China has recently become the second largest economy and is increasingly playing an important and influential role in global trade and infrastructure investment, particularly since the initiation of the Belt and Road Initiative in 2013 (National Development and Reform Commission (NDRC) 2018). In 2018, the World Bank ranked China 1st and the USA 2nd for GDP-PPP (gross domestic product taking into account purchasing power parity) (World Bank 2019). Further, China accounts for 18.5% of the world’s population and the USA an additional 4.3% (over one-fifth combined). Companies, products and employees from both countries are distributed across the world presenting opportunities for both importation and exportation of infectious diseases through animal products, human travel and wildlife trade, and as major drivers of ecological change responsible for the emergence of new diseases. China and the USA thus have imperative moral and fiscal responsibility to invest in global health security, and their cooperation is key for preparedness and control of global EIDs in the future.

The USA has several key government agencies which are actively contributing to emerging infectious disease research around the world including the Department of Defense, Health and Human Services, the President’s Emergency Plan for AIDS Relief, the President’s Malaria Initiative, the State Department and USAID [see program compilation available at the NIH Fogarty International Center website (National Institutes of Health Fogarty International Center 2019)]. The USA is currently the largest donor to global health in the world (approximately $11 billion in 2019); however, the current US administration has proposed significantly reducing global health funding for the fiscal year 2020 (to approximately $8 Billion) (Henry J Kaiser Family Foundation 2019). The USA also benefits from significant private investment in global health, and this trend is likely to continue, with efforts from private foundations such as the Gates Foundation and the Chan Zuckerberg Biohub poised to accompany US government funding of global health-related projects (Reubi 2018). China has also recently expanded its national infrastructure for EID preparedness and a country-wide network of laboratories including 11 national technology platforms, 11 national research centers and 6 national key laboratories. China also has several WHO collaborating centers with research focused on: tuberculosis, schistosomiasis, infectious disease surveillance, EIDs, management of HIV, influenza, vector surveillance, infectious disease epidemiology, echinococcosis, tropical diseases, malaria, and emerging and re-emerging infectious diseases (World Health Organization 2019b), and two OIE collaborating centers with research focused on food-borne parasites and zoonoses (World Organization for Animal Health 2019). A strong emphasis on leading edge technology for molecular diagnostics and pathogen characterization has made China a leader in the field of virology and biotechnology (Ellis 2018), including investment in the state-of-the-art pathogen isolation and identification technologies such as high-throughput sequencing. In January 2018, for the first time, China overtook the USA in terms of total number of scientific publications, according to statistics compiled by the US National Science Foundation (NSF) (Tollefson 2018).

**Figure 3:**
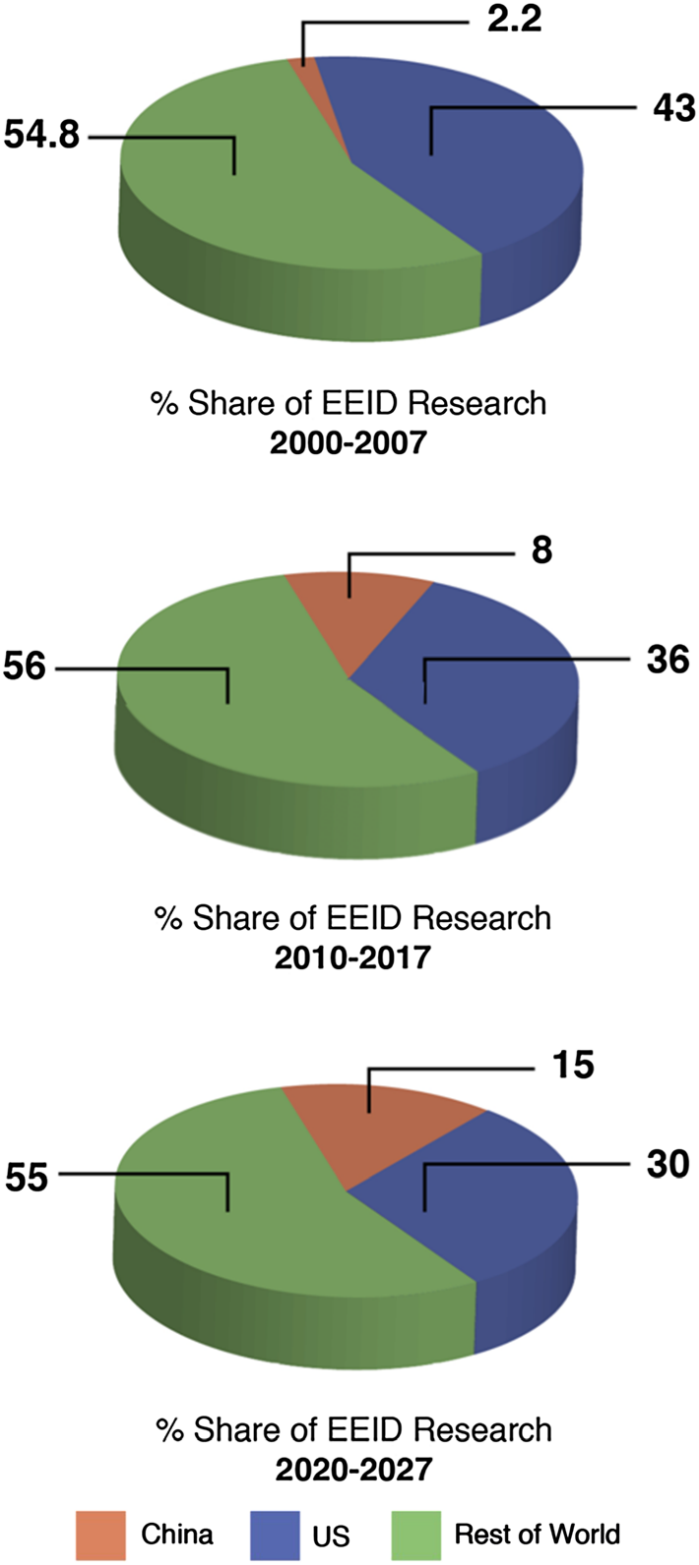
Current and projected future contributions to research involving the ecology and evolution of infectious diseases from China, the USA and the rest of the world based on the published literature.

Historically, China and the USA have placed different levels of emphasis on the ecological and evolutionary components of infectious diseases research. A search of the Web of Science (August 15, 2018: see supplementary information for details) revealed that from 2000–2007, 43% of publications on ecology/environment that included disease/pathogens were authored by US researchers exclusive of Chinese participation, while only 2.2% were authored by Chinese researchers exclusive of US participation. These figures changed to 36% and 8%, respectively, from 2010 to 2017. If this trend continues into the next decade (2020–2027), then under a linear extrapolation we can expect the USA and China to publish around 30% and 15% of ecological and evolutionary infectious disease-related research (Fig. 3). Together, the USA and China have published half of the world’s ecological and evolutionary infectious disease-related research. Despite these trends, and while around 45% of all published disease research is undertaken within the USA and China, integrated scientific studies with strong ecological and evolutionary components are largely missing.

## Proposed Next Steps

While current investments from the USA and China have built a foundation for better disease surveillance in humans and animals around the world, an investment in training in disease ecology with an emphasis on critical thinking remains a missing link. A key observation identified by us during the recent US–China workshops was that Chinese infectious disease research has a strong emphasis on biology, biotechnology, genomics, virology and public health surveillance at the expense of research into the spatial/geographic, social/cultural, transmission and ecological components needed to develop models to guide policies for controlling zoonoses and other outbreaks. This technology-oriented bias in research at the cost of a broader social and ecological understanding of disease systems can result in an intellectual trap where developing countries continue to depend on external ecological and environmental systems-level expertise critical to managing outbreaks. In addition, with the explosion of bioinformatics related research in the USA, it is important that the USA not lose sight of the fundamental importance of foundational training in ecological understanding. Future investments in training in the fields of disease ecology, eco-evolutionary dynamics, study design in natural systems, disease modeling and complex data analysis are essential for China—as it is no less for other Asian and sub-Saharan Africa countries—to take a lead role in their own disease investigations and to contribute toward global health security as equal partners.

An investment in human capacity for critical thinking regarding infectious diseases involves extending ecological and analytical training in less developed countries, where pathogens are most likely to emerge—moving beyond efforts limited to field sampling and pathogen detection. The UN China One Health Event held in 2011 emphasized the importance of problem-based as opposed to disciplinary learning to achieve these goals (Fearnley et al. 2019). This is the critical next step toward fully collaborative EID preparedness programs, so that capacity strengthening can be focused on preparing in-country teams for designing their own outbreak investigations, conducting their own human and wildlife disease ecology surveys, and taking a more active role at every step of the scientific process involved with understanding EIDs and responding to outbreaks. Significant challenges exist to accomplishing this holistic training approach, when in-country tertiary science education has not produced sufficient research groups able to carry out the needed research. China is the logical next step for the global health community’s major investment in disease ecology training, given its well-developed scientific infrastructure, current investments in basic science, virology and biotechnology, and national interest to contribute to global EID initiatives.

New approaches are needed to integrate ecological training and accelerate EID preparedness. These include broadening data collection to reduce uncertainties and improving analytical techniques to identify regions at highest risk for EIDs, as well as strengthening public health infrastructures in these locations to reduce the number of outbreaks. The application of novel analytical approaches to address these critical needs has been conflated by academic debate on whether or not EID prediction is possible (Geoghegan and Holmes 2017). While we currently do not have the ability to accurately forecast the time and location of the next EID outbreak, existing predictive models have been instrumental in prioritizing efforts. These include focusing on: sub-national regions (Allen et al. 2017; Jones et al. 2008); underlying environmental drivers (Allen et al. 2017; Johnson et al. 2015); specific reservoir host species (Olival et al. 2017); and pathogen traits (Olival et al. 2017; Fearnley et al. 2019) that facilitate spillover (Carlson et al. 2018). With a larger investment in disease ecology training in countries likely to be hot spots for EIDs, we can create the next generation of modeling and analytical techniques that incorporate more robust input from EID source nations.

Substantial decadal-long US–China joint funding mechanisms for integrated multidisciplinary EEID research projects are also needed to accomplish cross-training and meet research objectives discussed above. Efforts toward this mission have been limited because of unwillingness of traditional funders to redirect resources across sectors and expand out of siloed missions (Mazet et al. 2016). Disease-related research focused on medical or genetic components at the point of human infection and spread has received much more funding than that with an ecological health focus, aiming to describe the environmental or host community scenarios facilitating initial spillover of a pathogen to humans, or the impact of biodiversity loss on human health (Ostfeld 2017; Cardinale et al. 2012). An important step in the right direction was the Ecology and Evolution of Infectious Diseases (EEID) program through the NSF’s announcement in August 2018, of the addition of the National Natural Science Foundation of China as a new international collaborative partner. Through a relatively modest government investment of $275 million USD (including contributions from foreign partners) since 2000, this program has funded over 150 individual projects and led to some key discoveries that have greatly advanced our understanding and prediction of EID spillover, amplification and spread (Lloyd-Smith et al. 2005; Kilpatrick et al. 2006; Gilbert et al. 2008; Chiu et al. 2019; Lee et al. 2017; Carver et al. 2016; Coffey et al. 2008). Continuation of this program and further collaborative funding efforts between the USA and China are needed.

With the burgeoning world population and dramatically increased movements of individuals, the potential for a disease outbreak to cause the death of hundreds of millions of individuals is now a reality. Only a deep understanding of disease from an ecological systems point of view, taking into account every scale of a pathogen’s life cycle, can avert the increasing number of catastrophes in waiting. Very often it is information on disease ecology that is missing from programs purporting to take a One Health approach. Without the scientific and funding support of China and the USA in addressing the ecological components of disease systems through engagement of researchers and health practitioners from every part of the globe, we will continue to remain dangerously naive of how best to confront the threat of pandemic disease.

## Electronic Supplementary Material

Below is the link to the electronic supplementary material.

Supplementary material 1 (DOCX 18 kb)
