# Supplementary material for: Synergistic China–US Ecological Research is Essential for Global Emerging Infectious Disease Preparedness
Source: Ecohealth. 2020 Feb 3;17(1):160–73. doi: 10.1007/s10393-020-01471-2 (PMC7088356; doi:10.1007/s10393-020-01471-2)
Supplement: Supplementary file 1 — Supplementary material 1 (DOCX 18 kb) [file 10393_2020_1471_MOESM1_ESM.docx]

| **Subject Matter Topics and Timeframe** | **Total Number of Publications (Proportion)** | **USA number of publications (Proportion)** | **China number of publications (Proportion)** | **USA and China number of publications (Proportion)** | **Expected*** | **Expected / Observed**** |
| --- | --- | --- | --- | --- | --- | --- |
| Ecology & Disease & (2010-2018) | 6186 (1.0) | 2916 (0.471) | 295 (0.048) | 90 (0.015) | 0.022 | 1.55 |
| Ecology & Immunology & (2010-2018) | 291 (1.0) | 127 (0.436) | 6 (0.021) | 2 (0.007) | 0.009 | 1.31 |
| Ecology & Pathogen & (2010-2018) | 4389 (1.0) | 2065 (0.470) | 248 (0.057) | 79 (0.018) | 0.027 | 1.48 |
| Environment & Disease & (2010-2018) | 48020 (1.0) | 17257 (0.359) | 4157 (0.087) | 824 (0.017) | 0.031 | 1.81 |
| Environment & Pathogen & (2010-2018) | 21163 (1.0) | 7173 (0.339) | 1917 (0.091) | 359 (0.017) | 0.031 | 1.81 |
| Species model & Disease & (2010-2018) | 18330 (1.0) | 7263 (0.396) | 2250 (0.123) | 456 (0.025) | 0.049 | 1.96 |
| Species model & pathogen & (2010-2018) | 9303 (1.0) | 3817 (0.410) | 1050 (0.113) | 250 (0.027) | 0.046 | 1.72 |
| (Ecology OR Environment) & (Disease OR Pathogen) & (2000-2007)*** | 20541 (1.0) | 8783 (0.428) | 450 (0.022) | 97 (0.005) | 0.009 | 1.98 |
| (Ecology OR Environment) & (Disease OR Pathogen) & (2010-2017)*** | 61381 (1.0) | 22319 (0.364) | 4956 (0.081) | 1023 (0.017) | 0.029 | 1.76 |

Data represent a search of the Web of Science on August 15, 2018 for publications using the described subject matter topics and China and /or USA as author identifiers, published during the years specified.

*Under a random allocation of authorship model, if the proportions of papers on which USA and China are authors or coauthors in the same row are *x* and *y* respectively, then we expect the same row proportion on which both USA and China are authors to be *xy*. Thus, the expected level is the USA proportion in (column 3) multiplied by China proportion (column 4).

**This is the ratio of the previous entry in the same row (column 6) to the USA and China proportion in the same row (column 5).

***These time periods were chosen in order to select an 8-year period, during two consecutive decades, to predict, by linear extrapolation, contributions from China and the USA in 2020-2027.
